# Rural–Urban Disparities in Realized Spatial Access to General Practitioners, Orthopedic Surgeons, and Physiotherapists among People with Osteoarthritis in Alberta, Canada

**DOI:** 10.3390/ijerph19137706

**Published:** 2022-06-23

**Authors:** Xiaoxiao Liu, Judy E. Seidel, Terrence McDonald, Alka B. Patel, Nigel Waters, Stefania Bertazzon, Rizwan Shahid, Deborah A. Marshall

**Affiliations:** 1 Department of Community Health Science, Cumming School of Medicine, University of Calgary, Calgary, AB T2N 1N4, Canada; xiaoxili@ucalgary.ca (X.L.); judy.seidel@albertahealthservices.ca (J.E.S.); terrence.mcdonald@ucalgary.ca (T.M.); alka.patel@ahs.ca (A.B.P.); 2 McCaig Bone and Joint Health Institute, University of Calgary, Calgary, AB T2N 1N4, Canada; 3 O’Brien Institute for Public Health, University of Calgary, Calgary, AB T2N 1N4, Canada; bertazzs@ucalgary.ca (S.B.); rizwan.shahid@ahs.ca (R.S.); 4 Applied Research and Evaluation Services, Alberta Health Services, Edmonton, AB T5J 3E4, Canada; 5 Department of Family Medicine, Cumming School of Medicine, University of Calgary, Calgary, AB T2N 1N4, Canada; 6 Department of Geography, University of Calgary, Calgary, AB T2N 1N4, Canada; nwaters@ucalgary.ca; 7 Department of Civil Engineering, University of Calgary, Calgary, AB T2N 1N4, Canada; 8 Department of Environmental Science and Policy, College of Science, George Mason University, Fairfax, VA 22030, USA

**Keywords:** osteoarthritis, realized access, travel time, general practitioners, orthopedic surgeons, physiotherapists, distance decay, rural–urban

## Abstract

Rural Canadians have high health care needs due to high prevalence of osteoarthritis (OA) but lack access to care. Examining realized access to three types of providers (general practitioners (GPs), orthopedic surgeons (Ortho), and physiotherapists (PTs)) simultaneously helps identify gaps in access to needed OA care, inform accessibility assessment, and support health care resource allocation. Travel time from a patient’s postal code to the physician’s postal code was calculated using origin–destination network analysis. We applied descriptive statistics to summarize differences in travel time, hotspot analysis to explore geospatial patterns, and distance decay function to examine the travel pattern of health care utilization by urbanicity. The median travel time in Alberta was 11.6 min (IQR = 4.3–25.7) to GPs, 28.9 (IQR = 14.8–65.0) to Ortho, and 33.7 (IQR = 23.1–47.3) to PTs. We observed significant rural–urban disparities in realized access to GPs (2.9 and IQR = 0.0–92.1 in rural remote areas vs. 12.6 and IQR = 6.4–21.0 in metropolitan areas), Ortho (233.3 and IQR = 171.3–363.7 in rural remote areas vs. 21.3 and IQR = 14.0–29.3 in metropolitan areas), and PTs (62.4 and IQR = 0.0–232.1 in rural remote areas vs. 32.1 and IQR = 25.2–39.9 in metropolitan areas). We identified hotspots of realized access to all three types of providers in rural remote areas, where patients with OA tend to travel longer for health care. This study may provide insight on the choice of catchment size and the distance decay pattern of health care utilization for further studies on spatial accessibility.

## 1. Introduction

Osteoarthritis (OA) is a significant cause of pain and disability affecting 1 in 8 (13%) Canadians [1]. The prevalence of OA is expected to increase, reaching 1 in 4 Canadians by 2040 due to ageing population and increasing prevalence of obesity [2,3]. Although there is no known cure for OA, early treatment and management are critical to slow the disease progression, reduce symptoms, and improve quality of life [4]. The vast majority of OA care takes place in community-based clinics and by primary care physicians. Pain is the most common complaint among people with OA. Nonpharmacological therapy, which may involve treatment by a physiotherapist (PT)–allied health provider [5], is recommended for pain management and optimization of strength, flexibility, and function over the long term [4]. PTs typically provide treatment for OA in acute care hospitals, rehabilitation facilities, and within the community. Among the 2345 licensed PTs in 2013, approximately 47% of licensed PTs were in private practice compared to 29% in general hospitals [6]. In general, community-based physiotherapy is not an insured service and requires out-of-pocket costs and or copay by private insurance. People may visit a physiotherapist without a doctor’s referral. As the condition progresses, patients typically seek care from a general practitioner (GP) who may prescribe pharmaceuticals and/or lifestyle changes to manage the pain and improve function. Patients may also be referred by a GP or PT to other specialists, such as an orthopedic surgeon (Ortho), for more severe stages of the disease for surgical options [5]. Both primary care providers, orthopedic surgeons, and physiotherapists are key members of comprehensive team-based care with a family physician leadership that is patient-centred [7]. Patient-centred integrated care has been recommended in clinical practice guidelines with a goal of addressing health inequity and improving the quality of care by focusing on the needs and preferences of patients, especially for OA with a high prevalence of comorbidities [8]. In Alberta, which is unique in its focus on an integrated health care model to administer and deliver public health care to all Albertans, the Primary Care Alliance has proposed a transformed health care system with a vision to provide integrated primary, specialist, physiotherapist, and other community-based health care. Driven by population and community health needs, the integrated health system is aiming to address health inequity in quality of care, health outcomes, and access to care [8]. The government of Alberta initiated the Alberta Surgical Initiatives to reduce wait time to orthopedic surgeons through implementation, spread, and scale-up of centralized access and a triage referral system at the provincial level to improve access to specialty care [9].

Achieving equitable access to care is a priority at both national and provincial levels in Canada to reduce health disparities for patients in rural and remote areas [10,11]. In general, rural Canadians have higher health care needs compared to their urban counterparts due to higher prevalence of OA and comorbidities but less access to care due to the lack of health services in their home community [12]. It is reported that 21% of the Canadian population reside in rural areas, whereas only 9.4% of GPs and 3% of specialists [10,13] practice in rural areas. In Alberta, OA prevalence in rural remote areas was 135 cases per 1000 population, 26% higher than the prevalence in metropolitan areas (107 cases per 1000 population) [14]. People suffering from OA have a higher prevalence of comorbidities compared to the general population [15,16]. Among people with OA, the prevalence of 1, 2, and 3+ comorbidities present at the diagnosis of OA was observed to be 14%, 40%, and 23% higher than the prevalence in metropolitan areas, respectively [17]. Significant barriers to access may be associated with geography. An international comparative study reported that geographical barriers to accessing care with GP was more frequently reported in Canada compared to other countries (Australia, New Zealand, and Switzerland) between 2012 and 2014 [18]. Studies have documented the distance decay association between spatial access and health outcomes, that is, people who live in close vicinity of health care providers/facilities tend to have better health outcomes and increased health care utilization compared to those living further away [19,20].

Access to health care is multidimensional, with access barriers consisting of both spatial and aspatial dimensions [21]. The measure of spatial access can be broadly spilt into potential access and realized access [22]. Potential access refers to the availability of health services to potential users within a health care delivery system [23], which is usually measured with specialized gravity models such as the two-step floating catchment area method (2SFCA) [24,25,26] and the enhanced 2SFCA [27]. A key limitation in modeling spatial accessibility is the lack of available empirical data on the actual physician-seeking behavior [28,29,30,31] to verify model assumptions on the choice of reasonable catchment size as well as the distance decay pattern of health care utilization. Health care utilization patterns vary across population groups, health care providers, and the spectrum of diseases [23,32]. Although numerous studies on spatial access have been published for different populations (general population [27], immigrants [33], older population [31]), different health services and specialties (hospitalizations [32], medical clinics [34], primary care [27], specialty [35]) and different diseases (hypertension and diabetes [36]), few studies have examined the issue comprehensively among people with OA across health care providers. Realized access refers to the actual utilization of services by patients overcoming all barriers (e.g., language, physical disabilities, finance, and shortage of physicians) [10,37]. One measurement of a barrier or facilitator to realized access is the driving time or straight-line distance between a patient’s home address and the location of health services they utilize [22,23]. The measure of travel time and distance as a barrier or facilitator to access using empirical access behavior data is important to examine how far patients travel to seek health care and how travel distance and/or travel time interacts with health care utilization.

Examining realized access to three providers simultaneously provides a relatively comprehensive picture of gaps in access to needed OA care. It provides insight on the choice of catchment size and the distance decay pattern of health care utilization for accessibility assessment and health care resource allocation. It is important to provide information for improved identification of gaps in access to care that is provided by interprofessional teams and support evidence-informed decisions on coordinated and integrated care that is patient-centred [19]. In this study, we measured the realized access to three different types of health care providers for people with OA using utilized physician-seeking data. The aim of this study is three-fold: (1) to measure the rural–urban disparities in realized access to three types of health care providers (GPs, Ortho, and PTs); (2) to examine the spatial pattern of realized access at the local geographic area level; (3) to examine how the distance decay pattern of health care utilization varies along the rural-urban continuum.

## 2. Materials and Methods

Cross-sectional study design was applied to examine the rural–urban disparities in realized access to multidisciplinary health care providers for adult people with OA. An adult OA-prevalent cohort (≥18 years of age at diagnosis) in fiscal year 2013 (1 April 2012–31 March 2013) was identified using the most current and validated case definition for administrative data [3,14].

### 2.1. Data Sources

The health care services provided by GPs, Orthos, and PTs in fiscal year 2013 were of interest in this study. As community-based physiotherapy is not an insured service, this study only captured those publicly funded outpatient PT visits which were supported by the Alberta Health Care Insurance Plan (AHCIP). A prevalence cohort of 359,638 adult OA patients (≥18 years of age at diagnosis) in fiscal year 2013 was identified using the most current and validated case definition for administrative data [3,14]. We obtained administrative data from five Alberta Health (AH) administrative databases: AHCIP population registry, the Physician Claims Database (Claims), the Discharge Abstract Database, and the Ambulatory Care Classification System/National Ambulatory Care Reporting System. All the OA-prevalent patients had to meet the following two criteria: (1) became an OA patient in Alberta between 1994 and 2013; (2) did not migrate out of the province or die between 1994 and 2013 [3]. A detailed description of the case definition was reported previously [3,14]. Unique deidentified patient IDs were used to link patients with health records and health care providers.

We obtained the provider type and the six-digit postal code of their practice location from the Claims Database and the patients’ six-digit postal codes from the AHCIP population registry dataset. Patients were excluded if no health care was utilized for any of the three types of providers. Both patients and providers were geocoded using the 2013 Postal Code Translator File [38] created by AH.

### 2.2. Standard Geographic Areas

**Figure 1:**
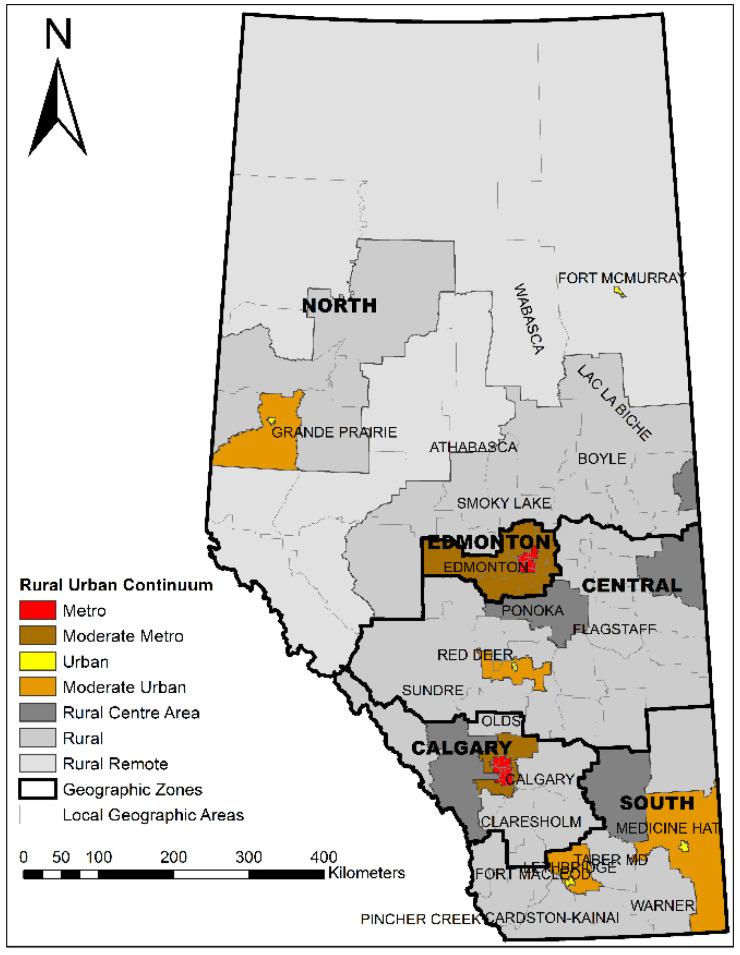
Standard geographic areas in Alberta.

A set of standard geographic areas was jointly created by Alberta Health Services (AHS) and AH for planning, surveillance, monitoring, and reporting of population health, health outcomes, and health services across Alberta [39] (Figure 1), including five zones (North, Edmonton, Central, Calgary, and South) and 132 local geographic areas (LGA)—the lowest geographic level in Alberta. Rural–urban continuum areas were further developed to analyze, report, plan, monitor, and compare population health by rural and urban status which grouped 132 LGAs into seven distinct categories (Metro, Moderate Metro influence, Urban, Moderate Urban influence, Rural Centre, Rural, and Rural Remote) [39].

### 2.3. Travel Time Calculations

Origin–destination network analysis was applied to calculate travel time in minutes from the origin of a patient’s postal code to the destination of the physician’s postal code. The travel time was calculated with the assumption that patients travelled by car under optimal conditions at posted speed limits, without delays due to rush hour, storms, road closures, detours, etc. AHS modeled a road network dataset from the DMTI Route Logistics Road Network [40] (2014), which classified roads into primary roads, secondary roads, and local roads. The Alberta Municipality Data Sharing Partnership (AMDSP) [41] road data (2013) were applied further to modify the DMTI road data and speed limits as AMDSP provided accurate speeds for the majority of municipalities and town in central and southern Alberta [42]. We adjusted the speed limits of road segments according to the AMDSP road data where they were available. Given the long winter time in Alberta, we applied a 20% reduction in the speed limit for travel distances of less than 50 km to obtain a conservative estimate of winter travel time [22]. Ferry routes, one-way roads, and trails were used as restrictions within the analysis. A snap distance of 5 km was applied to capture postal codes within 5 km of the roads. Time-based turn restrictions (such as prohibited left turn from 6:00 to 18:00) and delays at signals/stop signs were not included in the analysis as point data locations and delay times were not available. The road network was validated against the nonurgent emergency medical services (EMS) trips covering Alberta in 2013 [42], showing a difference of 0.26 min between the calculated travel time and the EMS travel time. Detailed information on analysis setting is available from the AHS document [42].

### 2.4. Descriptive Statistics

We grouped patients with OA by sex, age, rural–urban continuum, and type of health care providers (GPs, Orthos, and PTs). The numbers of patients/visits were presented for each subgroup. The percentage of patients/visits was calculated as the number of patients/visits within a subgroup divided by the number of patients/visits in Alberta. Boxplots of travel time in minutes by type of providers were provided to capture the distribution of travel times. Due to the positive skewness of travel time, as shown in Appendix A, the data on the boxplots were truncated at the 95th percentile [43] of travel time.

### 2.5. Hot Spot Analysis at the LGA Level

We wanted to determine if there was a spatial pattern in realized access at the LGA level to GPs, Orthos, and PTs among people with OA. Median travel time was used to represent the level of realized access within each LGA due to the skewed distribution of travel time values. Hot spot analysis based on the Getis-Ord Gi* statistic identified hot spots of realized access (LGAs with a significantly long median travel time surrounded by other LGAs with similar values) and cold spots of realized access (LGAs with a significantly short median travel time surrounded by other LGAs with similar values) [44,45,46]. The underlying null hypothesis of hot spot analysis was that the median travel time of each LGA followed a random distribution across Alberta. The alternative hypothesis was that it was nonrandom. Using the Getis-Ord Gi* routine, we calculated the Z-score and the P-value for each LGA to determine statistical significance of hot spots and cold spots [45]. The results of the hot spot analysis are presented in Section 3.4.

We captured the spatial interaction among neighboring LGAs using a spatial weights matrix that is based on the number of nearest neighbors or the distance between two LGAs [46]. Spatial weights are equally assigned to the LGAs within a defined neighborhood, indicating an equal influence on the LGA of interest [46]. We applied hot spot analysis using three different spatial weight matrices (eight nearest neighbors, fixed distance of 40 km with at least eight nearest neighbors, and queen’s continuity with at least eight nearest neighbors) to examine the sensitivity of the identified hot spots to different models of spatial interaction [17]. Detailed results are reported in Appendix B. Based on our previous study of OA prevalence patterns at the LGA level [17] as well as for maintaining consistency in the results of the hot spot analysis, we only reported results using the eight nearest neighbors spatial weights matrix.

### 2.6. Distance Decay Pattern of Health Care Utilization

Our null hypothesis was that there was no relationship between distance and health care utilization. Our alternative hypothesis was that there was a relationship and we tested a variety of alternatives represented by distance decay functions. The distance decay pattern depicted the principle that health care utilization decreases along with travel time. In this analysis, the cumulative probability approach [32,47] was applied to describe the distance decay behavior of health care visits to three types of providers along the rural–urban continuum. Health care visits were grouped by rural-urban status and then cumulated inversely by travel time in minutes. The distance decay function could be estimated by fitting a continuous relationship of the cumulative proportion of health care utilization with corresponding travel times, which was formulated as follows:

$$Y_{d}=\mu f\left(d\right)$$

where d is the travel time in minutes from a patient’s residential postal code to the provider’s practice postal code; $Y_{d}$ is the cumulative probability of health care utilization with the travel time greater than d; μ is a scalar; $f\left(d\right)\;$ is a set of three popular distance decay functions found in the literature, including exponential function ($e^{-\beta d})$, log-logistic function 1/1+d/θ^β,  and power function ($d^{-\beta })\;$[26,32]. Parameters β and θ are to be estimated for distance decay functions, indicating how fast the utilization of health care decreases with travel time.

The nonlinear least square estimator was used to estimate the parameters, and pseudo-R^2^ and the Akaike information criterion (AIC) were calculated to identify the best-fitting models [32]. To aggregate the number of health care visits to providers by travel time, we defined 68 travel time datapoints including 31 points within the 0–30 min range (every minute), 18 points from 31 to 120 min (every 5 min), 12 points from 121 to 240 min (every 10 min), six points from 241 min to 600 min (every 60 min), and the maximum (1 point). The model for Alberta and each rural–urban continuum had 68 observations to fit the distance decay functions.

Travel time calculation and hot spot analysis were conducted using ArcMap 10.8. Distance decay functions were estimated using R 4.0.2, the “aomisc” package. Ethics approval for this project was provided by the Conjoint Health Research Ethics Board at the University of Calgary (REB13-0100).

## 3. Results

### 3.1. Characteristics of Patients with OA in 2012/2013

Among the 359,638 patients with OA in 2012/2013, we identified 194,034 (54%) patients who visited at least one of the three types of providers. Of the 194,034 OA patients included in this analysis, 144,343 (74%) patients visited one type of providers, 35,781 (18%)—two, 13,190 (7%)—three types of providers. Among the 194,034 OA patients, 170,342 (88%) accounted for 577,899 GP visits (3.4 visits/patient), 47,370 (24%) patients—for 132,980 Ortho visits (2.8 visits/patient), and 39,923 (21%) patients—for 215,462 PT visits (5.4 visits/patient) (Table 1). The women utilized approximately 60% of the visits with each type of providers. The patients aged between 55–74 years accounted for approximately 50% of the visits with each type of health care providers. The patients residing in metropolitan areas accounted for 50% of the health care visits, compared to 20% in rural/remote areas. The number and percentage of patients and visits by provider type, age, sex, and comorbidities across the rural–urban continuums are shown in Appendix A.

### 3.2. Distribution of Patients and Providers

We identified 2312 GP practices (54% in metropolitan areas vs. 16% in rural/remote areas) accessed by the 170,342 patients residing in 45,614 postal code areas. We identified 243 Ortho practices which provided services to the 47,370 patients from 23,845 postal code areas. We identified 87 PT clinics (14 in metropolitan areas vs. 55 in rural/remote areas) that served the 39,923 patients from 20,039 postal code areas.

### 3.3. Travel Time in the Rural–Urban Continuum

**Figure 2:**
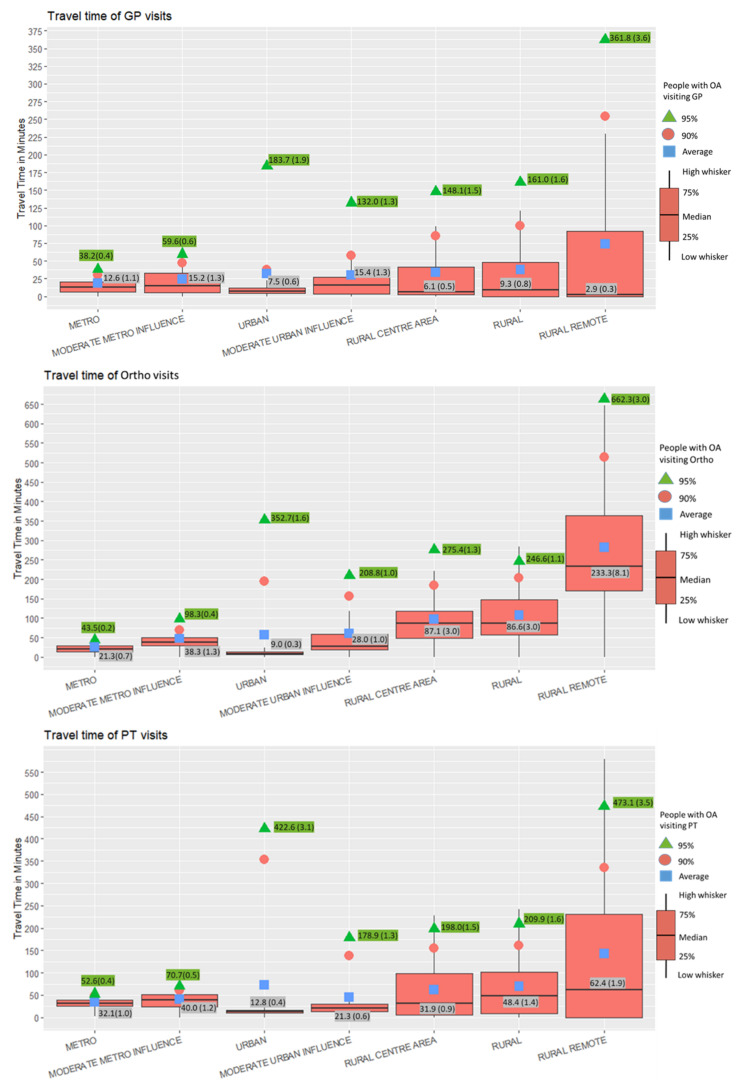
Boxplots of travel time to GPs, Ortho, and PTs along the rural–urban continuum. The numbers highlighted in grey denote the median travel time of each rural–urban category and the ratio to the Alberta median travel time. The numbers highlighted in green represent the 95th percentile of travel time within each rural–urban category and the ratio to the Alberta measure.

The median travel time in Alberta is 11.6 min (IQR = 4.3–25.7) to visit a GP, 28.9 min (IQR = 14.8–65.0) to visit an Ortho, and 33.7 min (IQR = 23.1–47.3) to visit a PT. A large variation in travel time to providers was observed along the rural–urban continuum (Figure 2). The median GP travel time was 2.9 min (IQR = 0.0–92.1) in rural remote areas, 77% shorter than in metropolitan areas (12.6; IQR = 6.4–21.0). The median Ortho travel time was 11 times higher in rural remote areas (233.3; IQR = 171.3–363.7) than in metropolitan areas (21.3; IQR = 14.0–29.3) and 26 times higher than in urban areas (9.0; IQR = 5.9–13.5). The median PT travel time is two times higher in rural remote areas (62.4; IQR = 0.0–232.1) than in metropolitan areas (32.1; IQR = 25.2–39.9) and five times higher than in urban areas (12.8; IQR = 9.8–16.9). More details are provided in Appendix B.

### 3.4. Hot Spots of Median Travel Time at the LGA Level

**Figure 3:**
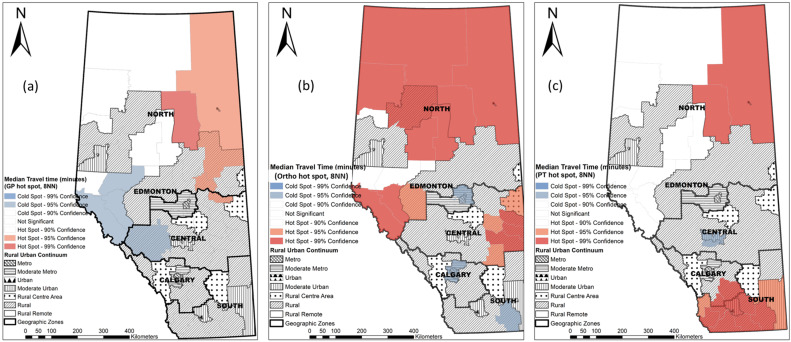
Hot spots of median travel time at the LGA level by primary practitioners (**a**), orthopedic surgeons (**b**), and physiotherapists (**c**).

The median travel time to a GP at the LGA level within the rural and remote areas is not homogeneously distributed (Figure 3a). The top 20% longest median travel times were observed mainly in northern remote, moderate metropolitan, and southwest rural areas. We identified hot spots of median travel time in northern rural remote areas and cold spots in western rural areas (Figure 3a).

The median travel time to an Ortho was shortest in urban and metropolitan areas, which increased along with the increase in rurality. We observed a 114.7-fold difference in travel time to an Ortho at the LGA level (4.2 min in urban Red Deer vs. 481.6 min in rural remote High Level). We identified nine hot spots in northern remote areas and five hot spots in eastern central rural areas. All the cold spots were identified in Metro-Edmonton and Metro-Calgary (Figure 3b).

Similarly, the median travel time to PT services was not evenly distributed within the rural and remote areas. Hot spot analysis identified three hot spots in northern rural remote areas, six hot spots in southern rural areas and the urban Lethbridge and the surrounding areas. All the cold spots were identified in the urban Red Deer and the surrounding areas (Figure 3c).

### 3.5. Distance Decay Pattern of Health Care Utilization

**Figure 4:**
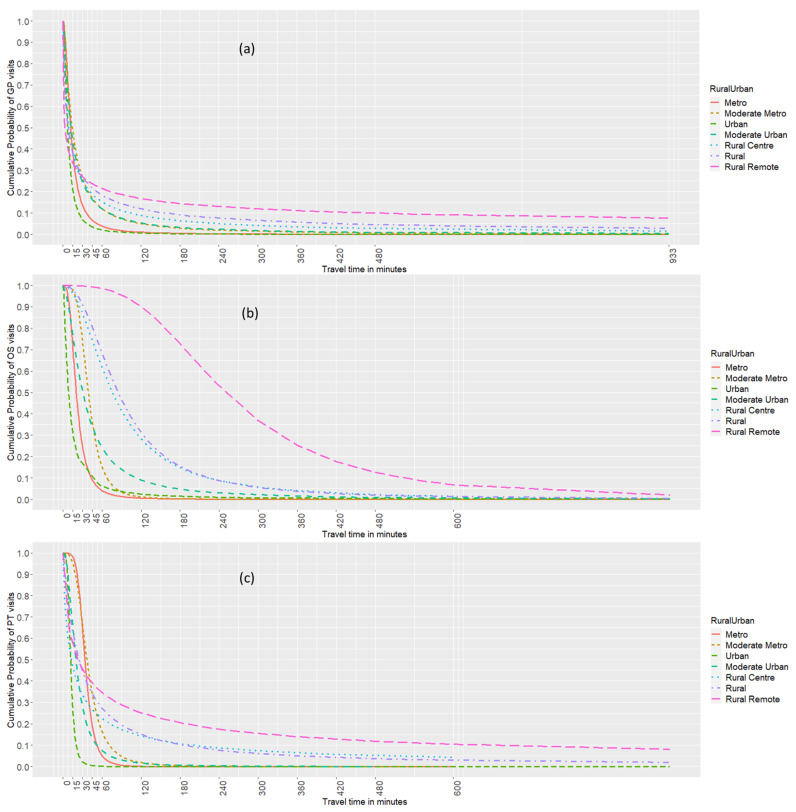
Log-logistic distance decay of health care visits by rural-urban continuum and health care providers—GP (**a**), Ortho (**b**), and PT (**c**).

The pseudo-R^2^ and AIC values for the three distance functions are reported in Table 2. The log-logistic function fitted best with higher pseudo-R^2^ and lower AIC values compared to the exponential function and the power function for most rural–urban groups in this study. As shown in Table 2, all parameters β and θ of the log-logistic models were statistically significant (*p* < 2 ∗ 10^−16^). These models produced low residual standard errors (RSE: 0.02–0.09 for GP visits; 0.01-0.09 for Ortho visits; 0.02–0.09 for PT visits) with a good curve fit [32,47] (Figure 4).

The distance decay patterns of health care utilization were captured by the fitted log-logistic functions, highlighting the association of increasing travel time with decreasing health care utilization. As shown in Figure 4a, most GP visits in urban/metropolitan areas occurred within 30 min from a patient’s residence (90%), whereas in rural/remote areas, the percentage dropped to 67%. Although 60% of the rural and remote patients travelled to a GP within 15 min, 24% travelled over 60 min. The remote patients showed the strongest distance decay effect within 15 min travel time, but also the weakest distance decay effect on GP visits when the travel time was longer than 30 min. As shown in Figure 4b, 90% of the patients in metropolitan and urban areas travelled up to 45 min for Ortho visits, which increased to 240 min in rural/rural centre areas and to about 510 min in remote areas. The effects of increasing travel time on Ortho visits were strongest in metropolitan/urban areas, while rural remote areas had the slowest decline in the number of Ortho visits with travel time. As shown in Figure 4c, urban areas had the strongest distance effects on PT visits with the steepest decline in the number of PT visits within 15 min travel time. Most patients had access to a PT within 60 min (90% in metropolitan areas, 70% in rural/rural centre, 60% in remote areas).

## 4. Discussion

Using administrative data, we observed significant rural–urban disparities in spatial access to health care providers along the rural–urban continuum. The rural remote patients had the shortest median travel time to visit a GP, while urban patients had the easiest access to an Ortho and a PT. Hot spot analysis revealed that northeastern rural remote areas experienced greatest geographic barriers to all the three types of providers. The rural remote patients were the least sensitive to the increase in travel time when seeking health care.

We observed that the median travel time to GPs in metropolitan areas was 12.6 min in Alberta, which is consistent with the literature. As reported in the literature, various travel threshold times to a GP, ranging from 9 to 30 min, have been used in the two-step floating catchment area method (2SFCA) [24,25,26] and other accessibility models, including the 11 min threshold to a GP in Toronto [30] and the maximum of 30 min catchment size in metropolitan Australia [48].

The significant rural–urban disparities in realized access to health care providers suggested that varying the catchment size may be required to capture the interaction between patients and health care providers. Catchment size delimits how far geographically services delivering health care to patients and at the same time determines how far patients travel to access the services. Distance and geographical isolation are the foremost health care access barriers [49,50]. As population dispersion increases, greater travel times are required to access nearby health care providers in more dispersed areas. Varying catchment size captures diverse travel patterns by rural–urban status and provides accurate estimates of access to care [48]. The results shed light on the selection of appropriate catchment sizes along the rural–-urban continuum, which will inform health care planners and policymakers on health resource allocation by defining health services areas based on travel time thresholds.

Our study utilized empirical data on actual physician-seeking behavior, which provided evidence on the choice of catchment sizes and distance decay association when modeling spatial accessibility. The measure of spatial accessibility can be broadly spilt into realized accessibility and potential accessibility [22]. Potential accessibility refers to potential availability of health services to potential users [19], which is usually measured with floating catchment area (FCA) metrics, such as the 2SFCA [24,25,26] and the enhanced 2SFCA [27]. To date, many applications of the FCA method have not been verified against empirical access behavior data [24,25,26]. A key limitation in modeling spatial accessibility is the lack of available empirical data on the actual physician-seeking behavior [28,29,30,31]. The FCA method assumes that patients visit the nearest health care facility within a specific catchment area, which ignores patient and system barriers to health care such as language, physical disabilities, and health literacy [10]. Furthermore, the FCA method requires assumptions of catchment size [33] and distance decay association between travel time/distance and health care utilization [32], which may vary by rural-urban status and type of health services [32,48]. However, the choice of reasonable catchment sizes and appropriate distance decay effect by rural–urban status and type of health services cannot be settled without empirical utilized health care data [30,31,48,51]. Our study sheds light on the key components of spatial accessibility modeling—choice of catchment sizes and distance decay effect on health care utilization, which may be applied to other regions that have a shortage of physicians in rural and remote areas due to misalignment with population health care needs.

We observed large variations in travel even within the same rural–urban continuum as shown in Figure 2 and Appendix B. Almost half of the health care visits to a GP in rural remote areas happened within the local postal code, while 95% of GP visits took up to 361.8 min. Urban areas had the shortest median travel time to an Ortho and a PT; however, 95% travelled up to 352.7 min to Ortho and up to 422.6 min to a PT, which was the second longest travel time to visit health care providers. As shown in the Figure 3, both the easiest (cold spot) and the most difficult (hot spot) access to a GP were identified in northern rural remote areas. The northern urban centre had significant geographic barriers to Ortho when compared to southern urban areas. Further studies at the local level as well as the individual level may help understand the most important factors driving this travel pattern.

The findings of the hot spot analysis shed light on health resource allocation to reduce rural–urban disparities in access to care. The government of Alberta initiated the Alberta Surgical Initiative in 2020 [52], emphasizing the importance of expanding telephone and electronic medical advice programs through which primary care providers can receive timely consultations from specialists for patients under care. Within northeastern remote areas where the patients had geographic barriers to all the three types of providers, health policies may be targeted to retain and recruit physicians and health care professionals (e.g., nurse practitioners and PTs) [53], as well as promote innovative methods of delivering health care (such as virtual care, TeleHealth, travel clinics, and integrated care) in remote areas [54,55]. For the northern remote areas that had a relatively easy access to GPs and PTs and, at the same time, a significantly difficult access to Ortho, integrated care may be targeted to increase Ortho capacity by having primary practitioners, PTs, and/or nurse practitioners to deliver timely advice to patients.

Physiotherapists play an integral role in optimizing OA patient outcomes before and after surgery [56,57], reducing health care costs [58], and increasing patient satisfaction [59]. However, it is challenging to evaluate rehabilitation services across Alberta due to variation in practice and funding resources [60]. The model of care was zone-specific. There was no provincial mechanism to collect, collate, and report data/outcomes related to physiotherapy services [60]. A previous study reported that reimbursement was linked to services rendered and the available human resources, showing that the ability or willingness to pay is a barrier to access to private community physiotherapy services in Alberta [61]. Health system efficiency and resource allocation are needed for rehabilitation services.

This study has numerous strengths. Firstly, we utilized empirical data to investigate the realized access to different health care providers within the rural–urban continuum for people with OA. This analysis is informative and will assist health planners and health service delivery by defining appropriate catchment sizes of health services, relocating health resources, and reducing the rural–urban disparities in access to health and health outcomes. Secondly, this study fills a gap in the modeling of spatial accessibility by providing evidence on the choice of catchment areas and distance decay effect on health care utilization along the rural–urban continuum. Thirdly, given the commonly reported outcomes of pain and reduced physical activity among people with OA, people with OA may present a different health care-seeking behavior as compared to other diseases [62]. Our study specifically focused on OA, providing evidence on the travel pattern of people with OA. Fourthly, we applied a validated algorithm based on administrative data to identify physiotherapy services that are coved by AHCIP. Lastly, we obtained the patients’ addresses at the six-digit postal code level from the administrative dataset, the finest spatial scale available for spatial analysis, which allows for the greatest detail regarding travel times.

We acknowledge the following limitations. Firstly, the travel time was calculated between the population-weighted centroids of two postal codes instead of geocoded addresses. However, due to privacy and confidentiality, six-digit postal codes at the patient level constitute the most detailed spatial scale for spatial analysis. Among the 57,874 postal codes identified with at least one OA case in Alberta, the average nearest distance between the postal codes ranged from 0.06 km in metropolitan/urban areas to 6.62 km in rural remote areas [14]. Given the large variation in the sizes of postal code areas, the travel time of rural patients visiting providers in their home postal code area may be longer compared to that of their urban counterparts. Secondly, the location and number of practices do not equal the number of providers. The capacity of each clinic was not accounted in this analysis. Thirdly, the travel time was calculated assuming optimal travel conditions. However, to make the best estimates, we utilized the impedance travel time which accounted for real-world conditions by taking a 20% reduction in speed limit [63]. Fourthly, the findings of the rural–urban disparities in access to OA care provide insights to the current health care delivery. Between 2014 and 2018, Alberta had fast growth in the number of doctors in urban Alberta (14% increase) compared to rural Alberta (6% increase). In particular, the number of doctors in rural Alberta in 2018 was down 3.6% from 2017. Though we saw a growth in the number of physicians in Alberta, physician service shortages continue in rural and remote areas of the province as well as in some underserved urban areas. Further, PT services in this study included only publicly funded outpatient visits, while those visits not supported by AHCIP were excluded. This study focused on the pattern of spatial access to three types of providers for people with OA. Confounding factors such as socioeconomic status on the pattern of realized travel time was not accounted for in this analysis due to unavailability of individual-level data. As a referral is required to visit an Ortho, the Ortho utilization pattern may be conditionally associated with the distribution and referral pattern of GPs. Lastly, as this analysis focused on the key providers relevant to OA management including GPs, Ortho, and PTs, patients who did not seek care from any of the three types of providers were not captured in this study. Further studies on the geospatial pattern of those OA patients not included in this analysis may be used as a weight for estimating the overall burden of geographic residence in assessing care.

## 5. Conclusions

This study examined the rural–urban disparities in realized access to health care providers for patients with OA. It is important to provide health planners with evidence on the thresholds of travel time to different health care providers along the rural–urban continuum. These findings may inform policies on accessibility evaluation and health resource allocation in Alberta. Further studies on the driving factors of travel patterns will be of interest. In the context of the COVID-19 pandemic where virtual care has been applied for many health services, the interpretation and meaning of “accessibility” may change accordingly. Further questions may be asked to answer to what extent the provision of virtual care may improve access to care for rural patients and what factors may affect equitable access to virtual care.

## Figures and Tables

**Table 1:** Characteristics of the patients with OA visiting general practitioners (GP), orthopedic surgeons (Ortho), and physiotherapists (PT) in 2012–2013.

Subgroups	GP Patients N (%)	GP Visits N (%)	Ortho Patients N (%)	Ortho Visits N (%)	PT Patients N (%)	PT Visits N (%)	OA Pop N (%)	Reg. Pop N (%)
Alberta	170,342 (100)	577,899 (100)	47,370 (100)	132,980 (100)	39,923 (100)	215,462 (100)	359,638 (100)	3,159,062 (100)
Sex								
Female	100,742 (59.1)	346,445 (59.9)	26,455 (55.8)	73,673 (55.4)	24,632 (61.7)	135,426 (62.9)	209,536 (58.3)	1,574,534 (49.8)
Male	64,862 (38.1)	211,450 (36.6)	19,871 (41.9)	56,140 (42.2)	14,504 (36.3)	76,609 (35.6)	150,102 (41.7)	1,584,528 (50.2)
Age groups (Years)								
18–34	2683 (1.6)	8769 (1.5)	1163 (2.5)	3889 (2.9)	0606 (1.5)	3248 (1.5)	21,939 (6.1)	1,056,429 (33.4)
35–44	8011 (4.7)	27,893 (4.8)	2104 (4.4)	6164 (4.6)	1300 (3.3)	6827 (3.2)	44,199 (12.3)	590,364 (18.7)
45–54	24,096 (14.1)	80,838 (14.0)	6698 (14.1)	18,585 (14.0)	4702 (11.8)	23,703 (11.0)	89,793 (25.0)	589,927 (18.7)
55–64	42,994 (25.2)	135,283 (23.4)	13,213 (27.9)	37,646 (28.3)	10,411 (26.1)	55,593 (25.8)	97,072 (27.0)	467,458 (14.8)
65–74	40,214 (23.6)	127,351 (22.0)	12,551 (26.5)	35,169 (26.4)	11,264 (28.2)	63,371 (29.4)	69,314 (19.3)	255,708 (8.1)
75–84	31,742 (18.6)	109,403 (18.9)	8324 (17.6)	22,558 (17.0)	8299 (20.8)	45,805 (21.3)	31,316 (8.7)	140,599 (4.5)
85+	15,864 (9.3)	68,358 (11.8)	2273 (4.8)	5802 (4.4)	2554 (6.4)	13,488 (6.3)	6005 (1.7)	58,577 (1.9)
Comorbidity								
No comorbidity	74,796 (43.9)	240,315 (41.6)	22,250 (47.0)	61,134 (46.0)	17,813 (44.6)	94,720 (44.0)	173,288 (48.2)	NA
One comorbidity	57,817 (33.9)	196,106 (33.9)	15,707 (33.2)	44,172 (33.2)	13,731 (34.4)	74,651 (34.6)	120,936 (33.6)	NA
Two comorbidities	24,150 (14.2)	87,429 (15.1)	6302 (13.3)	18,244 (13.7)	5613 (14.1)	31,723 (14.7)	47,909 (13.3)	NA
Three or more comorbidities	8841 (5.2)	34,045 (5.9)	2067 (4.4)	6263 (4.7)	1979 (5.0)	10,941 (5.1)	17,505 (4.9)	NA
Rural–urban continuum								
Metro	82,952 (48.7)	276,240 (47.8)	21,435 (45.3)	56,873 (42.8)	21,283 (53.3)	110,363 (51.2)	176,502 (49.1)	1,701,568 (53.9)
Moderate Metro	22,462 (13.2)	74,630 (12.9)	6200 (13.1)	16,655 (12.5)	7064 (17.7)	34,005 (15.8)	48,781 (13.6)	397,993 (12.6)
Urban	14,112 (8.3)	44,206 (7.6)	5916 (12.5)	20,020 (15.1)	1697 (4.3)	11,443 (5.3)	32,038 (8.9)	324,084 (10.3)
Moderate Urban	3287 (1.9)	9695 (1.7)	1183 (2.5)	3919 (2.9)	0368 (0.9)	2658 (1.2)	7389 (2.1)	65,344 (2.1)
Rural Centre	8334 (4.9)	31,342 (5.4)	2188 (4.6)	5992 (4.5)	1688 (4.2)	8010 (3.7)	16,399 (4.6)	130,994 (4.1)
Rural	34,575 (20.3)	123,373 (21.3)	9556 (20.2)	27,183 (20.4)	6890 (17.3)	41,449 (19.2)	70,408 (19.6)	469,086 (14.8)
Rural Remote	4620 (2.7)	18,413 (3.2)	0892 (1.9)	2338 (1.8)	0933 (2.3)	7534 (3.5)	8121 (2.3)	69,993 (2.2)

Note: N is the number of patients/visits by subgroups; % refers to the percentage of subgroup patients/visits among the Alberta population/visits; OA Pop denotes the OA-prevalent cohort identified between 1 April 2012 and 31 March 2013 using administrative data; Reg. Pop denotes the general population in Alberta that is obtained from the Alberta Health Care Insurance Plan population registry. Among the 170,342 patients who visited GPs, 4738 (2.8%) patients had missing information regarding age, sex, and comorbidities, accounting for 3.5% of the total GP visits. Among the 47,370 patients visiting Ortho, 1044 (2.2%) patients had missing information regarding age, sex, and comorbidities, accounting for 2.4% of the total Ortho visits. Among the 39,923 patients visiting PTs, 787 (2.0%) patients had missing information regarding age, sex, and comorbidities, accounting for 1.6% of the total PT visits.

**Table 2:** Distance decay associations between travel time and health care utilization.

GP Visits									
Rural–Urban Continuum	Exponential		Log-Logistic					Power	
	Pseudo-R^2^	AIC	Pseudo-R^2^	AIC	β	θ	RSE	Pseudo-R^2^	AIC
Alberta	0.998	−327.3	0.997	−317.8	1.27 ***	10.49 ***	0.02	0.936	−124.3
Metro	0.996	−294.6	0.998	−332.2	1.98 ***	11.82 ***	0.02	0.910	−83.4
Moderate Metro	0.996	−305.3	0.990	−226.7	1.39 ***	14.40 ***	0.04	0.914	−86.2
Urban	0.985	−196.1	0.991	−233.9	1.81 ***	7.21 ***	0.04	0.936	−140.4
Moderate Urban	0.996	−283.3	0.990	−233.4	1.26 ***	12.28 ***	0.04	NA	50.3
Rural Centre	0.971	−147.4	0.987	−245.3	0.88 ***	8.66 ***	0.04	0.956	−166.4
Rural	0.994	−319.5	0.949	−168.0	0.73 ***	7.87 ***	0.07	0.906	−130.7
Rural Remote	0.996	−364.8	0.834	−124.0	0.44 ***	3.11 ***	0.09	0.886	−156.0
**Ortho Visits**									
Rural–Urban Continuum	Exponential		Log-logistic					Power	
	Pseudo-R^2^	AIC	Pseudo-R^2^	AIC	β	θ	RSE	Pseudo-R^2^	AIC
Alberta	0.995	−247.0	0.999	−378.2	1.43 ***	29.16 ***	0.01	0.906	−63.4
Metro	0.985	−176.0	0.999	−346.5	2.94 ***	20.36 ***	0.02	0.867	−26.6
Moderate Metro	0.970	−103.9	0.999	−341.1	3.99 ***	38.82 ***	0.02	0.805	19.6
Urban	0.964	−92.9	0.958	−107.9	1.39 ***	8.45 ***	0.09	0.932	−103.3
Moderate Urban	0.980	−144.0	0.987	−172.7	1.68 ***	30.35 ***	0.06	−0.245	93.8
Rural Centre	0.981	−147.4	0.987	−151.7	2.04 ***	75.94 ***	0.07	0.810	−10.2
Rural	0.987	−180.4	0.998	−284.6	2.25 ***	84.55 ***	0.03	0.802	−4.1
Rural Remote	0.953	−61.9	0.998	−166.0	2.98 ***	250.18 ***	0.02	−0.146	90.3
**PT Visits**									
Rural–Urban Continuum	Exponential		Log-logistic					Power	
	Pseudo-R^2^	AIC	Pseudo-R^2^	AIC	β	θ	RSE	Pseudo-R^2^	AIC
Alberta	0.983	−164.4	0.995	−222.1	2.42 ***	31.67 ***	0.05	NA	111.2
Metro	0.962	−80.7	0.999	−324.0	5.02 ***	33.56 ***	0.02	0.157	125.6
Moderate Metro	0.975	−111.0	0.997	−258.0	3.33 ***	36.74 ***	0.03	0.807	16.6
Urban	0.961	−102.9	0.994	−170.7	3.93 ***	11.54 ***	0.05	0.873	−43.4
Moderate Urban	0.968	−103.2	0.978	−113.2	2.26 ***	19.67 ***	0.08	0.858	−26.9
Rural Centre	0.944	−140.6	0.975	−192.8	0.79 ***	12.75 ***	0.05	NA	59.0
Rural	0.995	−282.1	0.968	−151.2	1.08 ***	24.46 ***	0.08	0.866	−62.0
Rural Remote	0.964	−134.1	0.844	−67.3	0.66 ***	22.89 ***	0.09	−0.247	28.4

RSE: residual standard error; *** denotes statistical significance of parameters (*p* < 2 × 10^−16^).

**Figure A1:**
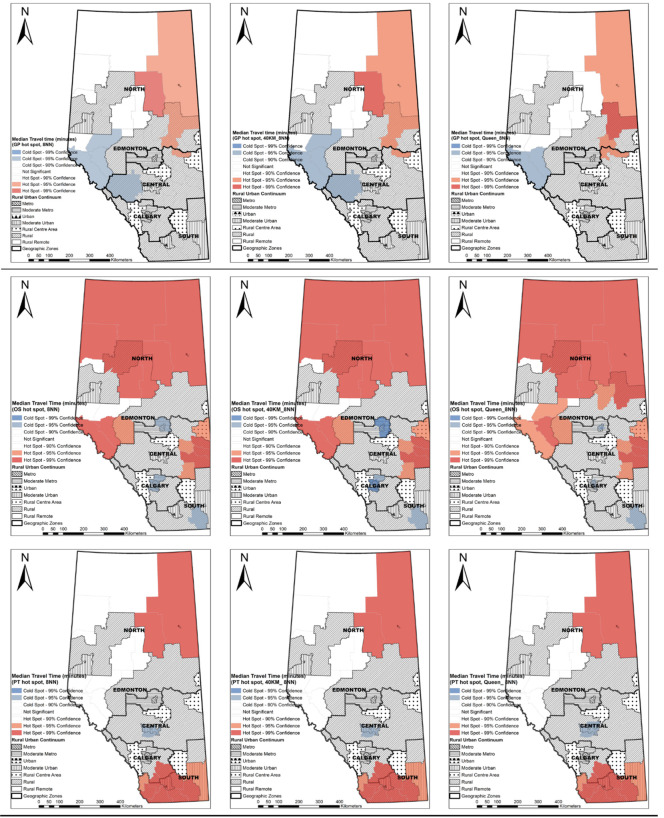
Hot Spot Analysis Using Three Different Spatial Weight Matrices.

## Data Availability

Restrictions apply to the availability of these data. Data was obtained from Alberta Health Services and are available from the corresponding author with the permission of Alberta Health Services.

## Appendix A

**Table A1:** Number and percentage of patients/visits by provider type, age, sex, and comorbidities across rural–urban continuum.

Providers	Subgroups	Metro N (%)	Moderate Metro N (%)	Urban N (%)	Moderate Urban N (%)	Rural Centre N (%)	Rural N (%)	Rural Remote N (%)	Alberta N (%)
**Number of patients by provider type, demographic factors, and rural–urban status**									
GP	Sex								
	Female	50,612 (50.2)	12,747 (12.7)	8361 (8.3)	1873 (1.9)	4799 (4.8)	19,683 (19.5)	2667 (2.6)	100,742 (100)
	Male	30,122 (46.4)	9134 (14.1)	5284 (8.1)	1341 (2.1)	3287 (5.1)	13,835 (21.3)	1859 (2.9)	64,862 (100)
	Age group (years)								
	18–34	1306 (48.7)	0330 (12.3)	0264 (9.8)	0068 (2.5)	0101 (3.8)	0497 (18.5)	0117 (4.4)	2683 (100)
	35–44	3742 (46.7)	0996 (12.4)	0713 (8.9)	0184 (2.3)	0461 (5.8)	1546 (19.3)	0369 (4.6)	8011 (100)
	45–54	11,502 (47.7)	3179 (13.2)	2147 (8.9)	0555 (2.3)	1213 (5.0)	4574 (19.0)	0926 (3.8)	24,096 (100)
	55–64	20,783 (48.3)	6124 (14.2)	3685 (8.6)	0871 (2.0)	2003 (4.7)	8322 (19.4)	1206 (2.8)	42,994 (100)
	65–74	18,946 (47.1)	5828 (14.5)	2903 (7.2)	0812 (2.0)	1937 (4.8)	8728 (21.7)	1060 (2.6)	40,214 (100)
	75–84	16,184 (51.0)	3742 (11.8)	2521 (7.9)	0516 (1.6)	1559 (4.9)	6615 (20.8)	0605 (1.9)	31,742 (100)
	85+	8271 (52.1)	1682 (10.6)	1412 (8.9)	0208 (1.3)	0812 (5.1)	3236 (20.4)	0243 (1.5)	15,864 (100)
	Comorbidity								
	No comorbidity	36,418 (48.7)	10,261 (13.7)	6164 (8.2)	1472 (2.0)	3491 (4.7)	15,163 (20.3)	1827 (2.4)	74,796 (100)
	One comorbidity	28,308 (49.0)	7752 (13.4)	4666 (8.1)	1127 (1.9)	2831 (4.9)	11,502 (19.9)	1631 (2.8)	57,817 (100)
	Two comorbidities	11,783 (48.8)	2900 (12.0)	2027 (8.4)	0449 (1.9)	1288 (5.3)	4919 (20.4)	0784 (3.2)	24,150 (100)
	Three or more comorbidities	4225 (47.8)	0968 (10.9)	0788 (8.9)	0166 (1.9)	0476 (5.4)	1934 (21.9)	0284 (3.2)	8841 (100)
Ortho	Sex								
	Female	12,534 (47.4)	3291 (12.4)	3261 (12.3)	0630 (2.4)	1157 (4.4)	5133 (19.4)	0449 (1.7)	26,455 (100)
	Male	8459 (42.6)	2784 (14.0)	2489 (12.5)	0527 (2.7)	0968 (4.9)	4220 (21.2)	0424 (2.1)	19,871 (100)
	Age group (years)								
	18–34	0605 (52.0)	0144 (12.4)	0144 (12.4)	0032 (2.8)	0041 (3.5)	0178 (15.3)	0019 (1.6)	1163 (100)
	35–44	0966 (45.9)	0282 (13.4)	0286 (13.6)	0057 (2.7)	0100 (4.8)	0347 (16.5)	0066 (3.1)	2104 (100)
	45–54	2954 (44.1)	0854 (12.8)	0892 (13.3)	0217 (3.2)	0320 (4.8)	1278 (19.1)	0183 (2.7)	6698 (100)
	55–64	5712 (43.2)	1885 (14.3)	1703 (12.9)	0348 (2.6)	0603 (4.6)	2687 (20.3)	0275 (2.1)	13,213 (100)
	65–74	5500 (43.8)	1793 (14.3)	1337 (10.7)	0305 (2.4)	0593 (4.7)	2824 (22.5)	0199 (1.6)	12,551 (100)
	75–84	4127 (49.6)	0909 (10.9)	1008 (12.1)	0167 (2.0)	0376 (4.5)	1622 (19.5)	0115 (1.4)	8324 (100)
	85+	1129 (49.7)	0208 (9.2)	0380 (16.7)	0031 (1.4)	0092 (4.0)	0417 (18.3)	0016 (0.7)	2273 (100)
	Comorbidity								
	No comorbidity	10,071 (45.3)	3000 (13.5)	2653 (11.9)	0558 (2.5)	1005 (4.5)	4577 (20.6)	0386 (1.7)	22,250 (100)
	One comorbidity	7186 (45.8)	2103 (13.4)	1914 (12.2)	0377 (2.4)	0707 (4.5)	3106 (19.8)	0314 (2.0)	15,707 (100)
	Two comorbidities	2848 (45.2)	0743 (11.8)	0864 (13.7)	0160 (2.5)	0320 (5.1)	1239 (19.7)	0128 (2.0)	6302 (100)
	Three or more comorbidities	0888 (43.0)	0229 (11.1)	0319 (15.4)	0062 (3.0)	0093 (4.5)	0431 (20.9)	0045 (2.2)	2067 (100)
PT	Sex								
	Female	13,567 (55.1)	4161 (16.9)	1022 (4.1)	0207 (0.8)	1023 (4.2)	4112 (16.7)	0540 (2.2)	24,632 (100)
	Male	7349 (50.7)	2763 (19.0)	0622 (4.3)	0148 (1.0)	0634 (4.4)	2608 (18.0)	0380 (2.6)	14,504 (100)
	Age group (Years)								
	18–34	0380 (62.7)	0088 (14.5)	0021 (3.5)	0008 (1.3)	0024 (4.0)	0074 (12.2)	0011 (1.8)	0606 (100)
	35–44	0732 (56.3)	0224 (17.2)	0048 (3.7)	0018 (1.4)	0057 (4.4)	0165 (12.7)	0056 (4.3)	1300 (100)
	45–54	2549 (54.2)	0845 (18.0)	0210 (4.5)	0049 (1.0)	0171 (3.6)	0726 (15.4)	0152 (3.2)	4702 (100)
	55–64	5483 (52.7)	1973 (19.0)	0476 (4.6)	0094 (0.9)	0407 (3.9)	1707 (16.4)	0271 (2.6)	10,411 (100)
	65–74	5682 (50.4)	2219 (19.7)	0396 (3.5)	0109 (1.0)	0524 (4.7)	2086 (18.5)	0248 (2.2)	11,264 (100)
	75–84	4621 (55.7)	1249 (15.1)	0354 (4.3)	0070 (0.8)	0366 (4.4)	1496 (18.0)	0143 (1.7)	8299 (100)
	85+	1469 (57.5)	0326 (12.8)	0139 (5.4)	0007 (0.3)	0108 (4.2)	0466 (18.2)	0039 (1.5)	2554 (100)
	Comorbidity								
	No comorbidity	9554 (53.6)	3250 (18.2)	0675 (3.8)	0158 (0.9)	0775 (4.4)	3023 (17.0)	0378 (2.1)	17,813 (100)
	One comorbidity	7411 (54.0)	2442 (17.8)	0567 (4.1)	0129 (0.9)	0548 (4.0)	2304 (16.8)	0330 (2.4)	13,731 (100)
	Two comorbidities	2960 (52.7)	0919 (16.4)	0282 (5.0)	0051 (0.9)	0251 (4.5)	0999 (17.8)	0151 (2.7)	5613 (100)
	Three or more comorbidities	0991 (50.1)	0313 (15.8)	0120 (6.1)	0017 (0.9)	0083 (4.2)	0394 (19.9)	0061 (3.1)	1979 (100)
**Number of visits by provider type, demographic factors, and rural–urban status**									
**GP**	**Sex**								
	Female	171,212 (49.4)	43,375 (12.5)	26,529 (7.7)	5620 (1.6)	18,398 (5.3)	70,381 (20.3)	10,930 (3.2)	346,445 (100)
	Male	95,730 (45.3)	28,755 (13.6)	15,960 (7.5)	3841 (1.8)	11,852 (5.6)	48,217 (22.8)	7095 (3.4)	211,450 (100)
	**Age group (years)**								
	18–34	3940 (44.9)	1125 (12.8)	0819 (9.3)	0176 (2.0)	0359 (4.1)	1932 (22.0)	0418 (4.8)	8769 (100)
	35–44	12,006 (43.0)	3269 (11.7)	2156 (7.7)	0581 (2.1)	1912 (6.9)	6355 (22.8)	1614 (5.8)	27,893 (100)
	45–54	37,155 (46.0)	10,082 (12.5)	6698 (8.3)	1533 (1.9)	4580 (5.7)	17,019 (21.1)	3771 (4.7)	80,838 (100)
	55–64	63,901 (47.2)	18,314 (13.5)	11,289 (8.3)	2520 (1.9)	7096 (5.2)	27,382 (20.2)	4781 (3.5)	135,283 (100)
	65–74	58,853 (46.2)	17,788 (14.0)	8398 (6.6)	2384 (1.9)	6774 (5.3)	29,050 (22.8)	4104 (3.2)	127,351 (100)
	75–84	54,683 (50.0)	13,474 (12.3)	7869 (7.2)	1505 (1.4)	5762 (5.3)	23,722 (21.7)	2388 (2.2)	109,403 (100)
	85+	36,404 (53.3)	8078 (11.8)	5260 (7.7)	0762 (1.1)	3767 (5.5)	13,138 (19.2)	0949 (1.4)	68,358 (100)
	**Comorbidity**								
	No comorbidity	113,701 (47.3)	32,105 (13.4)	18,247 (7.6)	4194 (1.7)	12,546 (5.2)	52,382 (21.8)	7140 (3.0)	240,315 (100)
	One comorbidity	95,008 (48.4)	25,566 (13.0)	14,620 (7.5)	3256 (1.7)	10,527 (5.4)	40,488 (20.6)	6641 (3.4)	196,106 (100)
	Two comorbidities	42,161 (48.2)	10,274 (11.8)	6818 (7.8)	1453 (1.7)	5286 (6.0)	18,373 (21.0)	3064 (3.5)	87,429 (100)
	Three or more comorbidities	16,072 (47.2)	4185 (12.3)	2804 (8.2)	0558 (1.6)	1891 (5.6)	7355 (21.6)	1180 (3.5)	34,045 (100)
**Ortho**	**Sex**								
	Female	33,217 (45.1)	8661 (11.8)	10,943 (14.9)	2062 (2.8)	3095 (4.2)	14,534 (19.7)	1161 (1.6)	73,673 (100)
	Male	22,461 (40.0)	7615 (13.6)	8407 (15.0)	1760 (3.1)	2708 (4.8)	12,072 (21.5)	1117 (2.0)	56,140 (100)
	**Age group (years)**								
	18–34	2028 (52.1)	0480 (12.3)	0500 (12.9)	0092 (2.4)	0129 (3.3)	0612 (15.7)	0048 (1.2)	3889 (100)
	35–44	2664 (43.2)	0889 (14.4)	0929 (15.1)	0164 (2.7)	0295 (4.8)	1065 (17.3)	0158 (2.6)	6164 (100)
	45–54	8027 (43.2)	2232 (12.0)	2866 (15.4)	0654 (3.5)	0884 (4.8)	3446 (18.5)	0476 (2.6)	18,585 (100)
	55–64	15,510 (41.2)	5068 (13.5)	5996 (15.9)	1174 (3.1)	1603 (4.3)	7516 (20.0)	0779 (2.1)	37,646 (100)
	65–74	14,336 (40.8)	4723 (13.4)	4493 (12.8)	1130 (3.2)	1638 (4.7)	8330 (23.7)	0519 (1.5)	35,169 (100)
	75–84	10,394 (46.1)	2397 (10.6)	3410 (15.1)	0502 (2.2)	1034 (4.6)	4550 (20.2)	0271 (1.2)	22,558 (100)
	85+	2719 (46.9)	0487 (8.4)	1156 (19.9)	0106 (1.8)	0220 (3.8)	1087 (18.7)	0027 (0.5)	5802 (100)
	**Comorbidity**								
	No comorbidity	26,228 (42.9)	7857 (12.9)	8601 (14.1)	1768 (2.9)	2723 (4.5)	13,011 (21.3)	0946 (1.5)	61,134 (100)
	One comorbidity	19,223 (43.5)	5727 (13.0)	6524 (14.8)	1304 (3.0)	1935 (4.4)	8617 (19.5)	0842 (1.9)	44,172 (100)
	Two comorbidities	7748 (42.5)	2012 (11.0)	2926 (16.0)	0570 (3.1)	0812 (4.5)	3818 (20.9)	0358 (2.0)	18,244 (100)
	Three or more comorbidities	2479 (39.6)	0680 (10.9)	1299 (20.7)	0180 (2.9)	0333 (5.3)	1160 (18.5)	0132 (2.1)	6263 (100)
**PT**	**Sex**								
	Female	71,764 (53.0)	20,401 (15.1)	7065 (5.2)	1421 (1.0)	5126 (3.8)	25,229 (18.6)	4420 (3.3)	135,426 (100)
	Male	37,033 (48.3)	13,017 (17.0)	4115 (5.4)	1171 (1.5)	2737 (3.6)	15,564 (20.3)	2972 (3.9)	76,609 (100)
	**Age group (years)**								
	18–34	2113 (65.1)	0424 (13.1)	0127 (3.9)	0038 (1.2)	0125 (3.8)	0361 (11.1)	0060 (1.8)	3248 (100)
	35–44	3491 (51.1)	1006 (14.7)	0287 (4.2)	0148 (2.2)	0321 (4.7)	1088 (15.9)	0486 (7.1)	6827 (100)
	45–54	11,964 (50.5)	3754 (15.8)	1216 (5.1)	0323 (1.4)	0789 (3.3)	4403 (18.6)	1254 (5.3)	23,703 (100)
	55–64	27,913 (50.2)	8997 (16.2)	3433 (6.2)	0851 (1.5)	1940 (3.5)	10,458 (18.8)	2001 (3.6)	55,593 (100)
	65–74	31,259 (49.3)	11,177 (17.6)	2933 (4.6)	0825 (1.3)	2515 (4.0)	12,675 (20.0)	1987 (3.1)	63,371 (100)
	75–84	24,663 (53.8)	6523 (14.2)	2393 (5.2)	0360 (0.8)	1779 (3.9)	8817 (19.2)	1270 (2.8)	45,805 (100)
	85+	7394 (54.8)	1537 (11.4)	0791 (5.9)	0047 (0.3)	0394 (2.9)	2991 (22.2)	0334 (2.5)	13,488 (100)
	**Comorbidity**								
	No comorbidity	48,109 (50.8)	15,245 (16.1)	4533 (4.8)	1161 (1.2)	3713 (3.9)	18,777 (19.8)	3182 (3.4)	94,720 (100)
	One comorbidity	39,058 (52.3)	12,049 (16.1)	3967 (5.3)	0967 (1.3)	2595 (3.5)	13,283 (17.8)	2732 (3.7)	74,651 (100)
	Two comorbidities	16,252 (51.2)	4514 (14.2)	2010 (6.3)	0329 (1.0)	1158 (3.7)	6339 (20.0)	1121 (3.5)	31,723 (100)
	Three or more comorbidities	5378 (49.2)	1610 (14.7)	0670 (6.1)	0135 (1.2)	0397 (3.6)	2394 (21.9)	0357 (3.3)	10,941 (100)

Note: N refers to the number of patients/visits; % in brackets refers to the percentage of subgroup OA patients within the total subgroup population in Alberta.

## Appendix B

**Table A2:** Summary statistics of travel time (minutes) by rural–urban continuum and types of providers.

	Rural–Urban Continuum	Average (Ratio to AB)	Minimum	P5	P25	Median	Median (Ratio to AB)	P75	P90	P95	Maximum
GP visit travel time	Metro	18.8 (0.7)	0.0	1.7	6.4	12.6	12.6 (1.1)	21.0	30.6	38.2	764.8
	Moderate Metro	24.4 (0.9)	0.0	1.3	5.5	15.2	15.2 (1.3)	33.1	47.4	59.6	803.3
	Urban	31.9 (1.2)	0.0	1.4	4.5	7.5	7.5 (0.6)	11.8	37.7	183.7	862.9
	Moderate Urban	29.8 (1.1)	0.0	0.0	3.8	15.4	15.4 (1.3)	27.4	57.7	132.0	798.7
	Rural Centre	33.8 (1.3)	0.0	0.0	2.8	6.1	6.1 (0.5)	41.1	85.9	148.1	837.4
	Rural	37.5 (1.4)	0.0	0.0	0.0	9.3	9.3 (0.8)	48.4	99.3	161.0	1162.2
	Rural Remote	74.3 (2.8)	0.0	0.0	0.0	2.9	2.9 (0.3)	92.1	254.4	361.8	1139.9
	Alberta	26.9 (1.0)	0.0	0.0	4.3	11.6	11.6 (1.0)	25.7	51.2	99.2	1162.2
Ortho visit travel time	Rural–urban continuum	Average (ratio to AB)	Minimum	P5	P25	Median	Median (ratio to AB)	P75	P90	P95	Maximum
	Metro	26.4 (0.5)	0.0	6.1	14.0	21.3	21.3 (0.7)	29.3	36.7	43.5	682.3
	Moderate Metro	47.6 (0.8)	0.0	20.7	29.5	38.3	38.3 (1.3)	50.2	70.2	98.3	685.5
	Urban	56.5 (1.0)	0.0	2.3	5.9	9.0	9.0 (0.3)	13.5	195.1	352.7	847.6
	Moderate Urban	60.2 (1.0)	0.0	11.0	18.0	28.0	28.0 (1.0)	58.2	156.0	208.8	626.2
	Rural Centre	96.9 (1.7)	0.0	3.5	48.4	87.1	87.1 (3.0)	118.2	184.1	275.4	555.9
	Rural	107.8 (1.8)	0.0	7.2	57.1	86.6	86.6 (3.0)	148.2	203.8	246.6	832.7
	Rural Remote	281.5 (4.8)	0.0	78.4	171.3	233.3	233.3 (8.1)	363.7	513.5	662.3	933.2
	Alberta	58.3 (1.0)	0.0	4.7	14.8	28.9	28.9 (1.0)	65.0	151.4	218.4	933.2
PT visit travel time	Rural–urban continuum	Average (ratio to AB)	Minimum	P5	P25	Median	Median (ratio to AB)	P75	P90	P95	Maximum
	Metro	33.8 (0.7)	0.0	11.6	25.2	32.1	32.1 (1.0)	39.9	48.0	52.6	558.7
	Moderate Metro	40.9 (0.9)	0.0	14.7	24.7	40.0	40.0 (1.2)	51.4	60.6	70.7	501.0
	Urban	72.3 (1.6)	0.0	4.6	9.8	12.8	12.8 (0.4)	16.9	353.7	422.6	669.7
	Moderate Urban	45.7 (1.0)	0.0	1.3	12.8	21.3	21.3 (0.6)	29.5	138.5	178.9	474.8
	Rural Centre	62.1 (1.4)	0.0	0.7	5.1	31.9	31.9 (0.9)	99.2	155.6	198.0	598.4
	Rural	69.3 (1.5)	0.0	0.0	8.7	48.4	48.4 (1.4)	102.1	161.9	209.9	870.7
	Rural Remote	143.2 (3.2)	0.0	0.0	0.0	62.4	62.4 (1.9)	232.1	335.9	473.1	903.7
	Alberta	45.3 (1.0)	0.0	3.0	23.1	33.7	33.7 (1.0)	47.3	80.4	135.3	903.7
